# Nursing care protocol for critical users with tracheostomy under mechanical ventilation

**DOI:** 10.1590/0034-7167-2023-0337

**Published:** 2024-05-27

**Authors:** Fernando Conceição de Lima, Wagner Felipe dos Santos Neves, André Lucas de Lima Dias, Clarissa Porfírio Mendes, Alzinei Simor, Ingrid Magali de Souza Pimentel, Helena Megumi Sonobe, Mary Elizabeth de Santana

**Affiliations:** I Universidade do Estado do Pará. Belém, Pará, Brazil; II Universidade de São Paulo. Ribeirão Preto, São Paulo, Brazil

**Keywords:** Nursing, Tracheostomy, Intensive Care Units, Practice Guideline, Education, Continuing, Enfermería, Traqueostomía, Unidades de Cuidados Intensivos, Guía de Práctica Clínica, Educación Continua, Enfermagem, Traqueostomia, Unidades de Terapia Intensiva, Guia de Prática Clínica, Educação Continuada

## Abstract

**Objectives:**

to develop and assess a nursing care protocol for critically ill users with tracheostomy under mechanical ventilation.

**Methods:**

a methodological study, developed through two phases, guided by the 5W2H management tool: I) target audience characterization and II) technology development.

**Results:**

thirty-four nursing professionals participated in this study, who presented educational demands in relation to care for critical users with tracheostomy, with an emphasis on standardizing care through a protocol and carrying out continuing education.

**Final Considerations:**

the creation and validity of new technologies aimed at this purpose enhanced the participation of nursing professionals and their empowerment in the health institution’s microsectoral actions and in macrosectoral actions, highlighting the need for public policies that guarantee the conduct of a line of care for users with tracheostomy.

## RESUMO

**Objetivos:**

desenvolver e avaliar um protocolo de cuidados de enfermagem para usuários críticos com traqueostomia em ventilação mecânica.

**Métodos:**

estudo metodológico, desenvolvido mediante duas fases, guiadas pela ferramenta gerencial 5W2H: I) caracterização do público-alvo e II) desenvolvimento da tecnologia.

**Resultados:**

participaram deste estudo 34 profissionais de enfermagem, que apresentaram demandas educacionais em relação aos cuidados com o usuário crítico com traqueostomia, com ênfase na padronização dos cuidados mediante um protocolo e realização de educação permanente.

**Considerações Finais:**

a criação e a validação de novas tecnologias voltadas para este fim potencializaram a participação dos profissionais de enfermagem e o seu empoderamento nas ações microssetoriais da instituição de saúde e nas ações macrossetoriais, por evidenciar a necessidade de políticas públicas que garantam a condução de uma linha de cuidado para usuários com traqueostomia.

## RESUMEN

**Objetivos:**

desarrollar y evaluar un protocolo de atención de enfermería a usuarios críticos con traqueostomía y ventilación mecánica.

**Métodos:**

estudio metodológico, desarrollado a través de dos fases, guiado por la herramienta de gestión 5W2H: I) caracterización del público objetivo y II) desarrollo tecnológico.

**Resultados:**

participaron de este estudio 34 profesionales de enfermería, quienes presentaron demandas educativas en relación al cuidado de usuarios críticos con traqueotomía, con énfasis en estandarizar los cuidados a través de un protocolo y realizar educación permanente.

**Consideraciones Finales:**

la creación y validación de nuevas tecnologías orientadas a este propósito han potenciado la participación de los profesionales de enfermería y su empoderamiento en las acciones microsectoriales y macrosectoriales de la institución de salud, por resaltar la necesidad de políticas públicas que garanticen la conducción de una línea de atención a usuarios con traqueotomía.

## INTRODUCTION

The rate of users undergoing tracheostomy (TCT), especially elderly men^(1)^, is considerably high, including in Intensive Care Units (ICUs)^(2)^, and therefore there must be an adequate level of knowledge among nursing professionals on care and management of complications to fill gaps in knowledge about adequate care for users living with TCT^(3)^.

Mechanical ventilation (MV) aims to maintain gas exchange, manage situations of hypoxemia and respiratory acidosis^(4)^. To achieve this, it is quite common to perform intubation with a cannula or tube via the orotracheal or nasotracheal routes. However, intubation can cause harm to users and, for this reason, it is recommended that users using orotracheal intubation (OTI) between 10 and 14 days, more than 6 hours a day and without interruption for 48 hours, be strongly indicated to perform TCT^(5, 6)^.

In ICUs, it is common for nursing professionals to develop care for users with TCT, since the reasons that lead to this reality are several, such as use of prolonged intermittent mandatory ventilation (IMV)^(6)^, airway protection, score on the Glasgow Coma Scale (GCS) less than 8, polyneuropathy, airway obstruction, favoring weaning from MV and offering comfort to users^(7)^.

Given the situation of performing TCT on behalf of IMV, it is necessary for a multidisciplinary team trained and qualified to provide the best service to users under such conditions, especially the nursing team, as they are responsible for care such as maintenance of airway patency, stoma and peristomal dressing, identification of complications and aspiration of users with TCT under IMV^(8, 9)^.

To this end, the need to train nursing professionals on assistance to users with TCT under IMV is emphasized, aiming to provide direct care to users, clinical assessment, effective interventions^(10)^ and adequate management of these devices in ICUs, reducing gaps important in the development of care practices^(11)^. In this way, the development of care protocols contributes to the standardization of care provided by nurses and the nursing team to users with TCT under IMV, as it allows for the Systematization of Nursing Care (SNC) provided, an essential aspect for quality and reduction risks related to stoma and its maintenance^(6)^.

## OBJECTIVES

To develop and assess a nursing care protocol for critical users with TCT under MV.

## METHODS

### Ethical aspects

The present study complied with all ethical aspects present in the development of research with human beings, as described in Resolution 466/2012 of the Brazilian National Health Council. The research project was approved by the Research Ethics Committee (REC) of *Hospital Ophir Loyola.*

### Study design, site and period

This is a methodological study^(12)^, carried out between March 2021 and November 2022, with development and assessment with the target audience of a care-assistance technology, in the format of a care protocol, called “care protocol for critical users with TCT”, in which the Revised Standards for Quality Improvement Reporting Excellence (SQUIRE 2.0) guidelines were followed. This study was carried out in a public hospital in the city of Belém, in the state of Pará, a reference in oncology in the North region.

### Population or sample; inclusion and exclusion criteria

Nursing professionals from the morning and afternoon shifts, working in ICUs of the aforementioned hospital, with at least one year’s experience in intensive care, were included. Nursing professionals who were on vacation or away from their institutional duties for any reason during the data collection period and who, for any reason, were unable to answer the questionnaires, were excluded.

The consecutive and non-probabilistic sample consisted of eight nurses and 26 nursing technicians. Professionals were invited to participate in the study voluntarily, after providing written consent by signing the Informed Consent Form (ICF), with one copy destined for participants and another that remained with the researchers.

### Study protocol

A methodological path was developed for developing this study, consisting of the phases: I) target audience characterization and II) technology development. To direct the phases, the 5W2H management tool was used, which contains seven action indicators, whose acronym contains the initials of processes in English, where: 1 - What; 2 - Who; 3 - When; 4 - Where; 5 - Why; 1 - How; 2 - How Much^(13)^. The option for this tool is justified by the possibility of mapping and operationalizing activities, as shown in Chart 1.

**Chart 1:** Action plan for developing a care protocol for users with tracheostomy under mechanical ventilation, Belém, Pará, Brazil

Phases	Action Plan
1. What (What will be done)	Development of TCT care protocol + clinical simulation activity
2. Who (Who)	Nurse/researcher
3. When (When)	September to October 2022
4. Where (Where)	5 ICUs of the aforementioned hospital
5. Why (Why it will be done)	SNC
6. How (How)	Carrying out an integrative review (IR)
7. How Much (Associated cost)	Internet access network, notebook, paper, ink and printer

*TCT – tracheostomy: ICU – Intensive Care Units; SNC – Systematization of Nursing Care.*

In the target audience characterization phase, aspects related to professional category, training time, professional qualification, length of service in the ICU, interest in training on TCT care, last training carried out on this topic and identification of the largest difficulty in carrying out TCT care were identified.

After analyzing nursing professionals’ profile and training needs, an IR was carried out, the protocol of which was registered on the figshare platform^(14)^, receiving the DOI number: https://doi.org/10.6084/m9.figshare.19566460.v2. In this process, the EndNote Web reference manager and the Rayann application were used^(15)^.

For product display and assessment, an educational activity was designed to train ICU nursing professionals based on the topics covered in the care protocol. To provide a welcoming environment for developing care skills in the face of updates to TCT care, a low-fidelity clinical simulation was developed using a mannequin to promote the development of skills and encourage the process of learning to learn. After data collection, nursing professionals were subjected to an activity assessment instrument to identify and analyze participants’ experience of the actions carried out by the researchers.

### Data analysis

Study data were described based on the gathering process during data collection. The information was synthesized, with theorization and transfer of findings, correlating them with IR data, which allowed their contextualization. Participant characterization in this research was carried out using simple descriptive statistics.

## RESULTS

### Target audience characterization

Target audience characterization indicated the participation of 34 nursing professionals, of which eight were nurses and 26 were nursing technicians. Regarding the training of these professionals, it is noted that they have been training and working in intensive care for more than 10 years. The characterization of these professionals and educational demands can be identified in Tables 1 and 2.

**Table 1:** Professional characterization of interviewed participants, Belém, Pará, Brazil

Professional characterization	Number of Answers
Number of professionals	34
Profession	
Nurse	8
Nursing technician	26
Specialization?	
No	22
Lato Sensu	12
Stricto Sensu (Master’s Degree)	3
Stricto Sensu (Doctoral Degree)	-
How Long Since Graduation	
1 to 4 years	-
3 to 5 years	6
6 to 10 years	5
More than 10 years	23
Time Working In The Intensive Care Unit	
1 to 2 years	5
3 to 5 years	9
6 to 10 years	6
More than 10 years	14

**Table 2:** Characterization of interviewed participants’ educational demands, Belém, Pará, Brazil

Educational demands	Number of Answers
Interest in TCT care	-
Yes	34
No	0
Training on TCT care	
No	19
Yes	15
<6 months	3
From 6 months to 1 year	10
More than 10 years	2
Importance of standardizing TCT care	
Yes	34
No	0
Importance of adopting a TCT care instrument in the service routine	
Yes	34
No	0
Importance of receiving continuing education in service	
Yes	34
No	0
Greatest difficulties in relation to TCT care	-
Identify emergencies and complications related to TCT	9
Mobilize secretions	6
Perform TCT aspiration	5
Dressing and peristomal skin care	5
Cuff Care	8
Changing the TCT tube	9
Carrying out nursing diagnoses (if a nurse)	3
Communication with users	16
None	2

In relation to educational demands, nursing professionals pointed out the interest and need to deepen their knowledge in relation to the care of critical users with TCT; all 34 interviewees showed an interest in standardizing the care provided to critical users with TCT through a care protocol and carrying out continuing education actions. The greatest difficulties faced by nursing professionals were focused on identifying emergencies and complications related to TCT (nine), in addition to communicating with users (16) and changing TCT tube (nine).

### Development of technologies

A care protocol-type technology was developed, the elaboration of which began with the process of illustration and composition of the content found in IR. The editing and layout phases continued, following the stages of structure/organization, layout, language, design, cultural adaptation sensitive to the public.

The care protocol was prepared in portrait format, divided into 13 parts. The cover of the protocol contains a presentation of the information entitled “Care protocol for users with TCT under mechanical ventilation” and authorship credits. On the remaining 12 pages, the content topics were organized, covering the following topics: general care; what are the possible emergencies and complications related to TCT?; secretion mobilization; when and how to perform TCT aspiration?; what should I know to perform good dressing and care for peristomal skin?; what care should I take with the cuff?; changing TCT tube - when is the ideal time?; nursing diagnoses for users with TCT; communication as an instrument of care and continuing education for the health team, people with TCT and caregivers. In the care protocol design, visual resources were applied to attract readers’ attention to the most pertinent information, making reading stimulating for the target audience. Font size and style were chosen to facilitate effortless and adapted reading, but without compromising the quality of information content.

To make this possible, an IR was carried out, and as a result, a final sample was obtained consisting of four studies^(16, 17, 18, 19)^, which assessed the emerging data, which were categorized into I) health care nursing with TCT and II) quality management of users with TCT. Furthermore, with the results of IR, it was indicated the need to make the discharge process standardized, in addition to standardizing TCT management care and the importance of professional training to enable TCT care, supported by evidence to the proposal of a care protocol.

### Skills training

For the skills training carried out with nursing professionals, six steps were followed: planning; learning objectives; training structure and format; setting description and skills training; 5. briefing; 6. feedback.

### Planning

Training planning for nursing professionals was carried out in four phases: I) Determination of needs and target audience: prior contact was made with the target audience (nursing professionals) to map their interest in participating in the research. This interest was identified through a questionnaire containing closed-ended questions; at this stage, it was also possible to map professionals’ prior knowledge about the key points of research, in addition to knowing the difficulties related to TCT management due to IMV, identifying participants’ professional and educational profile, and an invitation to the clinical simulation activity was sent. II) Theoretical framework: provided by IR findings, which supported the simulated activity based on the best scientific evidence on the topic. III) Simulated activity facilitator: This phase was carried out by a resident nurse in the second year of the intensive care residency and a preceptor, master’s degree holder and professor, who has significant knowledge on the proposed topic and adopted simulation experience. IV) Learning assessment strategy: it was decided to use a questionnaire produced by the authors themselves so that the target audience could assess the positive points, points for improvement and suggestions in relation to the clinical simulation carried out and the care protocol delivered and, thus, obtain tangible subsidies to continue producing the technology with future instrument validity and sharing in the health collection with the community in general.

### Learning objectives; Skill training structure and format; Setting description

Learning objectives were established according to the researchers’ planning, described previously, requiring nursing professionals to acquire skills to act in a humanistic, critical and reflective manner based on the care needs of critical users with TCT due to IMV, in addition to qualification for the exercise of nursing practices based on scientific and intellectual rigor based on ethical principles. They must provide nursing care to critical users with TCT by IMV according to their technical competence, since it is a category composed of nursing technicians and nurses and the development of skills of the technical-scientific training type that confers quality to professional practice, appropriately using the care protocol proposed for nursing practice.

To encourage learning and interaction, an instrument was made available, built by the researchers, with the aim of understanding and discussing aspects related to care for critical users with TCT due to IMV. A care protocol was then proposed, as it offers an effective method, with easy access, taking into account infrastructure limitations. A protocol must have a brief communication format, which provides understanding of content by consulting its information based on scientific evidence.

The skills acquisition activity was carried out in five ICUs, two of which served clinical oncology users and three for surgical oncology users and kidney transplant recipients. To develop the activity, pre-scheduling was carried out with the teams, so as not to interfere with professionals’ service routine. The hospital does not have a continuing education service, and as it is an intensive care environment, there was no possibility of relocating professionals. Therefore, the activity was developed in the sector itself (on-site) to include the entire nursing team present willing to participate.

### Briefing

After obtaining the final product, this study was planned in three dynamic moments to carry out skills training. Firstly, taking into account participants’ comfort in relation to skills training, a conversation circle was held, with prior exploration of participants’ knowledge so that the moment could be carried out horizontally, with the perspective that all participants felt comfortable and responsible for the learning process.

After socializing participants’ prior knowledge, theorization and transfer of information regarding experiences based on skills training in relation to TCT by IMV were carried out, clarifying the rules, objectives and theoretical frameworks, aiming for participants to achieve the proposed learning objectives.

In the second moment, of skills training itself, using a mannequin-type prototype, which simulates the cervical and chest region, training could be carried out, favoring significant learning. With the simulated activity, techniques for correct TCT aspiration, peristomal region cleaning and dressing, peristomal skin care, in addition to changing TCT fixation tape and discussion of topics present in the care protocol described previously were carried out, which reinforces the skills and abilities of each nursing team member in caring for users with TCT via MV, as it is a category composed, in the study, of nurses and nursing technicians who have specific tasks.

### Feedback

At the end of the activity, in the third moment, participants answered an assessment form of the activities carried out called “how nice”, “how about” and “what a shame” so that researchers could have feedback from participants about the activity developed, becoming aware of the strengths and points for the improvement process for greater effectiveness and meeting nursing professionals’ needs^(20)^.

**Figure 1:**
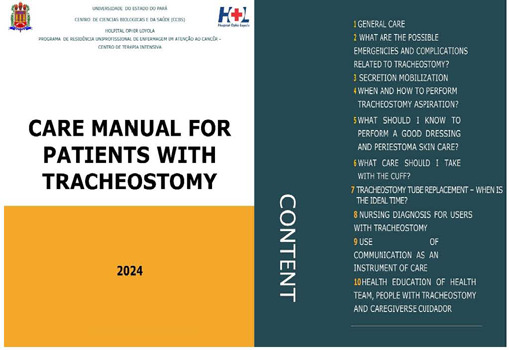
Technologies used in the validity and training stages, Belém, Pará, Brazil

The target audience’s assessment in relation to technologies produced allowed the analysis and contemplation of technological production execution and planning to guide their process of future optimization and to adapt to nursing professionals’ educational and care demands. Participants highlighted the weaknesses and strengths of technologies developed (educational activity and care protocol), highlighting important aspects and information. The categorization and synthesis of these reports are in Chart 2, the final version of the care protocol is available on the SciELO Data platform, with the cover and summary contained in Figure 1.

**Chart 2:** Important aspects related to the weaknesses and strengths found in the statements about the proposed technologies, Belém, Pará, Brazil

Item assessed	How nice	What a shame	How about
Educational activity	- “That there was health education in the service about TCT care”. - “It clarified some doubts about tracheostomized user care, so we can offer more efficient and safe care”. - “That a dynamic was carried out to fix the content”.	- “That there is no constant continuing education”. - “That not the entire team participated” - “That carrying out the service’s care activities hindered my participation in all moments of the educational action”.	- “If it were offered to a larger audience and in an auditorium”. - “Extending this action to the wards and other ICU shifts and professionals”.
Protocol	- “That it provides updated knowledge to improve our care”. - “That it provides clear and accessible language”.	- “That there is no institutionalized protocol for tracheostomized user care”.	- “Adding illustrations to the material”. - “Developing care protocols for other types of users”.

*TCT – tracheostomy: ICU – Intensive Care Units.*

## DISCUSSION

With the findings, it was demonstrated that nursing care in the ICU is essential for critical user rehabilitation, emphasized by professionals on the importance of the entire team’s participation during the exposure of technologies for development, with joint action of knowledge. Formal and informal structures were created to manage demands, collaboratively, in line with comprehensive and resolute assistance, which presupposes actions of respect and support^(21, 22)^. Thus, the concepts of hierarchy and subordination were balanced in favor of improving care, with articulation of knowledge, actions and services.

Knowing the nursing team’s educational needs and profile has been essential for planning the care provided as well as for appreciation and satisfaction of the institution’s nursing professionals, such as continuing education, quality management and ombudsman sectors, which constitute key elements for integration and optimization of the assistance provided^(3)^. Therefore, this study was concerned with consulting, integrating and meeting professionals’ educational demands in the process of developing the care protocol. Furthermore, a theoretical-practical educational activity was carried out to expose the care included in this care protocol.

It was identified that the majority of professionals have trained and worked in the ICU for more than 10 years, without specialization in the intensive care area, depending exclusively on updates and knowledge offered by the hospital. Therefore, the health institution must adopt actions and measures focused on continuing education for practical assistance training, through activities that promote the acquisition of specific and qualified knowledge from the perspective of comprehensive care^(3)^.

Regarding professionals’ knowledge and educational demands, it was revealed that the greatest difficulties in relation to TCT care were communication with users using TCT, changing the TCT tube, identifying emergency situations and cuff care, which constitute basic aspects of care for this clientele. However, these precautions have been the greatest obstacles in assisting critical users, as incorrect execution can lead to clinical complications for users^(10)^. Therefore, continuing education on handling the device and stoma becomes essential for quality management and user safety^(23, 24)^.

The need to improve knowledge and assistance provided to clients was evident, however there is no center for the development of continuing health education. The on-site difficulties identified were the lack of interest and participation of employees in the slightest attempts at updating, work overload, lack of infrastructure and suitable places to carry out educational activities, similar to other studies^(25, 26)^.

Another study with 254 physicians and nurses from four large hospitals identified that professionals had more difficulties related to adequate cuff pressure, emergency care, such as tube obstruction, adequate time for removal of TCT fixation points and signs of stoma infection^(23)^.

Recent studies in the area support the findings of this study, as professionals yearn for institutional standardization to improve TCT care, with a view to reducing complications and improving results in care for critical users with TCT^(7, 11)^. In this scenario, care protocols and educational technologies are allied tools in the process of professional training and care quality management. In this study, it was found that participants were eager for this standardization and use of a protocol to prevent complications related to stoma and device use^(27)^.

These instruments, with a care-educational focus, have been mentioned by authorities as possibilities for empowering those who use them, and in hospitals, they allow reconstructing concepts and valuing professionals’ experience for critical-reflexive, autonomous and change-generating action in the care context^(28)^. With this, the protocols benefit, instrumentalize and reorient the implementation of knowledge and care in relation to users with TCT, contributing to SNC^(6)^.

It is noteworthy that the target audience’s participation in the care protocol development and assessment, in meeting their educational needs, favors interaction between the multidisciplinary team, improvement of the assistance provided as well as recognition of the role of each of these actors in the care process. The inclusion of professionals in the instrument construction brought the desired result closer, as they were responsible for informing about the needs identified in the material^(29)^. As for the photographic expressions deemed insufficient, they were modified and sent to a professional to handle images’ layout and expressions. It should be noted that each item has its value in the instrument creation process so that the final product is appropriate for the target audience^(30)^. Visual identity allows effective communication and has important ethical, moral and social value, correlating its content with the social imaginary to achieve greater purpose through messages, symbols and signs^(31)^.

It was noted that the proposed technologies were innovative in the context of scientific production on this topic. Thus, they will be able to support future studies and contribute to nursing professionals’ clinical practice and continuing education and the establishment of a line of care to assist people with ostomies. Technological innovation interferes with the lives of those who use it, as it drives transformation in life and work. It is related to the theoretical conception of idealizing a solution to a problem by proposing, testing, building and adjusting its content, influencing the strengthening of continuing education actions and care management^(32)^.

In this study, the need for continuing education on TCT care beyond ICUs was identified by participants, which can contribute to legitimate institutionalization of continuing education practice in the hospital environment and generate benefits for professionals. It is emphasized that these actions were linked to knowledge innovation, meaningful learning, user and professional safety in their care actions, quality of care and user satisfaction^(33)^.

### Study limitations

As limitations of this study, the lack of content validity by expert judges, appearance validity by design judges, semantic validity by the target audience and usability assessment were identified; however, with the strong aspect of continuity projects, this project went through all these stages.

Furthermore, it contributed to increased research of this nature and theme, in addition to serving as motivation for developing other research that carefully assesses the processes of applying technologies through continuity projects, with the purpose of monitoring, in the short, medium and long term, the repercussions on the health team. Furthermore, the absence of public policies that highlight a specific line of care for users with TCT signals the need for technological tools as a strategy for continuing education and assertive assistance, through using validated and institutionalized technologies.

### Contributions to nursing, health or public policy

The care protocol was a relevant technology for mediating individual and group care practices in an organized manner, addressing topics considered most inconsistent by professionals in assisting users with TCT in intensive care.

## FINAL CONSIDERATIONS

The instrument construction and application with the target audience made it possible to: identify the professional profile as well as the nursing team’s educational interest that deals directly with users with TCT due to IMV, even in the face of the fragility of the educational activities offered by the hospital; perceive real interests in educational activities aimed at the entire team and in institutionalized instruments that systematize nursing care; highlight the most prevalent gaps in participants’ knowledge on the topic; and demonstrate that approaching the target audience in this construction process, listening to praise, criticism and contributions, reaffirms the importance of each individual in the process of learning to learn, through participatory and meaningful knowledge.

The method adopted supported the process of construction and rapprochement with participants, including the development of other technologies, both on this topic and others, which directly involve the educational need in care practice. It is noteworthy that the need to create and validate new technologies aimed at this purpose enhanced the participation of nursing professionals and their empowerment in the health institution’s microsectoral actions and in macrosectoral actions, for highlighting the need for public policies that guarantee the conduction of a line of care for users with TCT.

## Supplementary Material

0034-7167-reben-77-02-e20230337-suppl01

## Data Availability

https://doi.org/10.48331/scielodata.DVPU02
