# Supplementary material for: Nursing care protocol for critical users with tracheostomy under mechanical ventilation
Source: Rev Bras Enferm. 2024 May 27;77(2):e20230337. doi: 10.1590/0034-7167-2023-0337 (PMC11135913; doi:10.1590/0034-7167-2023-0337)
Supplement: 0034-7167-reben-77-02-e20230337-suppl01 [file 0034-7167-reben-77-02-e20230337-suppl01.pdf]

UNIVERSIDADE DO ESTADO DO PARÁ

CENTRO DE CIENCIAS BIOLOGICAS E DA SAÚDE (CCBS)

HOLPITAL OPHIR LOYOLA

PROGRAMA DE RESIDENCIA UNIPROFISSIONAL DE ENFERMAGEM EM ATENÇÃO AO CANCEÊR - CENTRO DE TERAPIA INTENSIVA

PROTOCOLO DE CUIDADOS COM TRAQUEOSTOMIA POR VENTILAÇÃO MECÂNICA

2022

APRESENTAÇÃO

Caro profissional de enfermagem,

ESTE MANUAL FOI ELABORADO COM O INTUITO DE CONTRIBUIR COM A EDUCAÇÃO EM SERVIÇO DA EQUIPE DE ENFERMAGEM DO CENTRO DE TERAPIA ITENSIVA DO HOSPITAL OPHIR LOYOLA, MEDIANTE AS ATUALIZAÇÕES EM RELAÇÃO AO CUIDADO DE PACIENTES CRÍTICOS COM TRAQUEOSTOMIA.

CONTEÚDO

1 CUIDADOS GERAIS

2 QUAIS AS POSSÍVEIS EMERGÊNCIAS E COMPLICAÇÕES RELACIONADAS À TRAQUEOSTOMIA?

3 MOBILIZANDO SECREÇÕES

4 QUANDO E COMO REALIZAR A ASPIRAÇÃO DA TRAQUEOSTOMIA?

5 O QUE EU DEVO SABER PARA REALIZAR UM BOM CURATIVO E CUIDADOS COM A PELE PERIESTOMA?

6 QUAIS CUIDADOS DEVO TER COM O CUFF?

7 CUIDADOS COM A CÂNULA DE TRAQUEOSTOMIA METÁLICA

8 TROCA DO TUBO DE TRAQUEOSTOMIA – QUAL O MOMENTO IDEAL?

9 DIAGNÓSTICOS DE ENFERMAGEM AO USUÁRIO COM TRAQUEOSTOMIA

10 A COMUNICAÇÃO COMO INSTRUMENTO DE CUIDADO

11 EDUCAÇÃO EM SERVIÇO DA EQUIPE DE SAÚDE, PESSOA COM TRAQUEOSTOMIA E CUIDADOR

1

CUIDADOS GERAIS

Quais cuidados tomar durante a higiene oral do paciente?

- Realizar higiene oral com digluconato de clorexidina 0,12% de 12-12 horas ou enxaguante bucal.

Como devo evitar a broncoaspiração?

- Mantendo a cabeceira do leito elevada entre 35º e 45º;
- Interrompendo a dieta por cateter de dubbhoff ou cateter de levine durante higiene bucal;
- Realizando aspiração da secreção durante o banho de aspersão ou banho no leito.

Atenção

- Ofertar oxigênioterapia conforme prescrição médica
- Posicionar adequadamente o circuito do ventilador sem que haja retrações ou retorno de água do condensado para o tubo
- Avaliar a posição da cânula
- Avaliar e realizar controle da dor se necessário.

Antes de iniciar os procedimentos, atente-se ao uso dos EPIS e da higienização das mãos

2

QUAIS AS POSSÍVEIS EMERGÊNCIAS E COMPLICAÇÕES RELACIONADAS À TRAQUEOSTOMIA

É necessário saber reconhecer as possíveis emergências e complicações que podem ocorrem decorrente da confeccção da traqueostomia, como:

- Pequenas hemorragias;
- Sangramento periestomal
- Posicionamento incorreto da cânula
- Enfisema subcutâneo
- Traqueomalácia
- Tosse
- Oclusão da cânula por secreção
- Disfagia e Disfonia
- Broncoaspiração
- Infecção
- Escape de ar

Antes de iniciar os procedimentos, atente-se ao uso dos EPIS e da higienização das mãos

3

MOBILIZANDO SECREÇÕES

A mobilização da secreção pode ser realizada seguindo três etapas:

I – Promover uma hidratação adequada
II – estimular a mobilidade física
III – remover as secreções (nebulizar e humidificar o ar com sf 0,9%, se possível utilizar trocadores de umidade do tipo HME, gás quente, peça em T ou máscara de traqueostomia.

Antes de iniciar os procedimentos, atente-se ao uso dos EPIS e da higienização das mãos

4

QUANDO E COMO REALIZAR A ASPIRAÇÃO DA TRAQUEOOSTOMIA

Quando realizar a aspiração da traqueostomia?

Atenção que a aspiração da traqueostomia só deve ser realizada se realmente for necessário e o usuário apresentar alguns sinais, como:

- Dispnéia (<12MRP)
- Uso de musculatura acessória
- Baixa saturação de Oxigênio (SPO2: <92%)
- Cianose
- Ausculta pulmonar com ruídos adventícios (Roncos, estertores, sibilos)
- Presença de secreção ao redor da traqueostomia
- Denteamento da curva de fluxo (se em uso de ventilação mecânica)

E como realizar o procedimento?

- Nebulizar e pré-oxigenar antes do procedimento por 30-60 segundos
- Aspirar cuidadosamente para não ocasionar lesão traqueal
- Não utilizar SF 0,9% direto na traqueostomia para aspirar
- Manter pressão de aspiração <120mmHg e > 100mmHg
- Utilizar cateteres de múltiplos lúmens
- Aspirar somente na retirada do cateter por no máximo 15 segundos e somente três vezes
- Estimular exercício de tosse
- E registrar as características das secreções drenadas

Antes de iniciar os procedimentos, atente-se ao uso dos EPIS e da higienização das mãos

5

O QUE EU DEVO SABER PARA REALIZAR UM BOM CURATIVO E CUIDADOS COM A PELE PERIESTOMA?

O tipo de curativo e os cuidados que serão realizados irão variar de acordo com a avaliação do profissional, logo é importante:

- Inspecionar e registrar a aparência da pele ao redor do estoma em busca de sinais de infecção e/ou irritação
- Realizar o curativo com SF 0,9% + Clorexidine aquosa, no mínimo uma vez ao dia
- Realizar a troca do cadarço de fixação uma vez por turno e sempre que necessário, atentando-se para que ele esteja sempre limpo e seco
- O cadarço deve ser de velcro ou algodão
- Manter no máximo dois dedos de folga antes de fixar o cadarço
- Priorizar a utilização de dois cadarços para uma boa fixação
- Utilizar gazes inteiras nas laterais da cânula (sem cortar e evitar gazes com fiapos) ou utilizar placas de hidrocolóide
- Manter a pele limpa, seca e hidratada para evitar infecção, maceração e lesão.

Antes de iniciar os procedimentos, atente-se ao uso dos EPIS e da higienização das mãos

6

QUAIS CUIDADOS DEVO TER COM O CUFF?

- o ideal é avaliar a pressão do cuff utilizando o cuffmetro e manter a pressão entre 20-30cm de H20.

Antes de iniciar os procedimentos, atente-se ao uso dos EPIS e da higienização das mãos

7

CUIDADOS COM A CÂNULA DE TRAQUEOSTOMIA METÁLICA

Os cuidados com a cânula de traqueostomia metálica se diferem, em parte, dos que são realizados com a cânula plástica, como:

- A frequência da limpeza deve ser de acordo com a necessidade do usuário e presença de secreção
- A limpeza da cânula e da endocânula deve ser realizada, se possível, com água morna e corrente, desde que a água possua aspecto transparente e livre de resíduos
- Utilizar escovinha traqueal para auxiliar na remoção de secreção
- Utilizar sabão neutro
- Após a limpeza da cânula, secar bem antes de reposiciona-la

Antes de iniciar os procedimentos, atente-se ao uso dos EPIS e da higienização das mãos

8

TROCA DO TUBO DE TRAQUEOSTOMIA QUAL O MOMENTO IDEAL?

O Procedimento deve ser realizado por profissional treinado

Para realizar a troca do tubo, seja plástico ou metálico, deve-se

- **utilizar uma cânula com ¾ do diâmetro traqueal do paciente**
- **Suspender alimentação por 3 – 4 horas antes do procedimento**
- **Deve ser realizado de sete a dez dias da realização da traqueostomia e se for metálica trocar a cânula externa a cada 30 dias.**

Antes de iniciar os procedimentos, atente-se ao uso dos EPIS e da higienização das mãos

9

DIAGNÓSTICOS DE ENFERMAGEM AO USUÁRIO COM TRAQUEOSTOMIA

Os diagnósticos de enfermagem à pessoa com traqueostomia auxiliam à tomada de decisão profissional, o cuidado isento de erros, a qualidade da assistencia e a satisfação do usuário.

- Comunicação verbal prejudicada
- Interação social prejudicada
- Nutrição desequilibrada menos do que as necessidades corporais
- Troca de gazes prejudicada; padrão respiratório ineficaz
- Ventilação espontanea prejudicada
- Integridade da pele prejudicada
- Risco de aspiração e Risco de infecção
- Desobstrução ineficaz das vias aéreas

Antes de iniciar os procedimentos, atente-se ao uso dos EPIS e da higienização das mãos

10

O USO DA COMUNICAÇÃO COMO INSTRUMENTO DE CUIDADO

É importante manter e criar alternativas de comunicação que possibilite expressar mesagens de forma escrita, não verbal e pela decodificação das espressões.

- A comunicação deve ser eficaz, de maneira fácil, acessível, humanizada, acolhedora e educativa
- Deve-se encorajar a equipe de saúde e acompanhantes a manter e estimular a comunicação e procurar fazer perguntas fechadas
- A comunicação, se bem feita, pode identifcar as necessidades do paciente, informar e esclarecer sobre os procedimentos ou necessidades que ele deseja saber.

Antes de iniciar os procedimentos, atente-se ao uso dos EPIS e da higienização das mãos

11

EDUCAÇÃO EM SAÚDE COM A EQUIPE DE SAÚDE, PESSOA COM TRAQUEOSTOMIA E CUIDADOR

A educação em saúde é uma ferramenta que valoriza os contextos sociais, econômicos e culturais dos usuários, cuidadores e profissionais, aliados ao processo de promoção da saúde. É um cuidado fundamental que a equipe de saúde deve adotar como atribuição diária.

A educação deve ser permanente e o treinamento eficaz para evitar e reduzir chances de complicações e permitir o reconhecimento das emergências e complicações relacionadas à traqueostomia.

Antes de iniciar os procedimentos, atente-se ao uso dos EPIS e da higienização das mãos

UNIVERSIDADE DO ESTADO DO PARÁ

CENTRO DE CIENCIAS BIOLOGICAS E DA SAÚDE (CCBS)

HOLPITAL OPHIR LOYOLA

PROGRAMA DE RESIDENCIA UNIPROFISSIONAL DE ENFERMAGEM EM ATENÇÃO AO CANCÊR - CENTRO DE TERAPIA INTENSIVA

ORGANIZADORES:
ENF. RESIDENTE. FERNANDO LIMA
PROF DRA MARY ELIZABETH DE SANTANA
ENF. WAGNER FELIPE NEVES
